# Functional Connectivity Disruption in Frail Older Adults Without Global Cognitive Deficits

**DOI:** 10.3389/fmed.2020.00322

**Published:** 2020-07-08

**Authors:** Isabel Suárez-Méndez, Sandra Doval, Stefan Walter, Natalia Pasquín, Raquel Bernabé, Ernesto Castillo Gallo, Myriam Valdés, Fernando Maestú, David López-Sanz, Leocadio Rodríguez-Mañas

**Affiliations:** ^1^Laboratory of Cognitive and Computational Neuroscience (UCM-UPM), Center for Biomedical Technology (CTB), Technical University of Madrid (UPM), Madrid, Spain; ^2^Department of Structure of Matter, Thermal Physics and Electronics, Complutense University of Madrid (UCM), Madrid, Spain; ^3^Department of Experimental Psychology, Complutense University of Madrid (UCM), Madrid, Spain; ^4^Foundation for Biomedical Research, University Hospital of Getafe, Madrid, Spain; ^5^Centro de Investigación Biomédica en Red Fragilidad y Envejecimiento Saludable (CIBERFES), Madrid, Spain; ^6^Department of Medicine and Public Health, Rey Juan Carlos University, Madrid, Spain; ^7^Radiology Service, University Hospital of Getafe, Madrid, Spain; ^8^Geriatric Service, University Hospital of Getafe, Madrid, Spain; ^9^Centro de Investigación Biomédica en Red en Bioingeniería, Biomateriales y Nanomedicina (CIBER-BBN), Madrid, Spain; ^10^Department of Psychobiology, Faculty of Psychology, Complutense University of Madrid (UCM), Madrid, Spain

**Keywords:** frailty, magnetoencephalography, functional connectivity, resting-state networks, aging

## Abstract

Frailty is a common representation of cumulative age-related decline that may precede disability in older adults. In our study, we used magnetoencephalography (MEG) to explore the existence of abnormalities in the synchronization patterns of frail individuals without global cognitive impairment. Fifty-four older (≥70 years) and cognitively healthy (Mini-Mental State Examination ≥24) adults, 34 robust (not a single positive Fried criterion) and 20 frail (≥3 positive Fried criteria) underwent a resting-state MEG recording and a T1-weighted magnetic resonance imaging scan. Seed-based functional connectivity (FC) analyses were used to explore group differences in the synchronization of fronto-parietal areas relevant to motor function. Additionally, we performed group comparisons of intra-network FC for key resting-state networks such as the sensorimotor, fronto-parietal, default mode, and attentional (dorsal and ventral) networks. Frail participants exhibited reduced FC between posterior regions of the parietal cortex (bilateral supramarginal gyrus, right superior parietal lobe, and right angular gyrus) and widespread clusters spanning mainly fronto-parietal regions. Frail participants also demonstrated reduced intra-network FC within the fronto-parietal, ventral attentional, and posterior default mode networks. All the FC results concerned the upper beta band, a frequency range classically linked to motor function. Overall, our findings reveal the existence of abnormalities in the synchronization patterns of frail individuals within central structures important for accurate motor control. This study suggests that alterations in brain connectivity might contribute to some motor impairments associated with frailty.

## Introduction

Population aging arises due to the increase in life expectancy, and the sharp reduction of birth rates. In this context, *frailty* represents a highly prevalent expression of pathological aging (1). This construct is commonly used to describe a multifactorial geriatric syndrome that involves a general worsening in health status prompted by decreased homoeostatic reserves and exacerbated vulnerability upon stressors. Even though a definitive consensus on the definition of frailty is still lacking (2, 3), Fried's phenotype is a validated and commonly used instrument in the clinical assessment of frailty (4, 5). The diagnosis of frailty according to Fried requires that at least three of the following physical manifestations are met: unintentional weight loss, decreases in gait speed and/or grip strength, self-reported exhaustion, and low energy expenditure (4).

The involvement of the central nervous system (CNS) in frailty constitutes a controversial topic, fueled in part by the unresolved link between frailty and cognitive impairment (2, 6–8). Surprisingly enough, only a few studies have looked into the neural basis of frailty *as an entity*, addressing the specific structural, physiological, and functional implications of the syndrome (9–13). Instead, several authors have focused on the relationship between brain pathology and individual presentations of frailty, such as gait speed and grip strength [(14, 15), reviewed in (16)]. This indirect evidence provides a relevant yet insufficient frame for the characterization of the neural correlates of the syndrome. As a consequence, the role of the CNS in frailty still represents a wide gap in current geriatric literature (16).

Functional connectivity (FC) measures reveal the statistical dependencies between the activity patterns of anatomically separated brain regions (17) and have been successfully applied to the study of brain communication in healthy and pathological aging (18–20). Up to date, only one publication has investigated the link between FC and frailty. Using functional Magnetic Resonance Imaging (fMRI), Lammers et al. reported decreases in FC in frail individuals, particularly affecting motor areas (21). Complementary research came from reports in healthy samples, which explored the FC patterns that subserve some motor features known to deteriorate with frailty. For example, it was found that increased FC in the frontal (FN), sensorimotor (SMN), and fronto-parietal (FPN) networks was associated with gait mechanisms (22), while FC between the dorsal attentional network (DAN) and the default mode network (DMN) influenced gait variability (23).

On the other hand, certain brain areas are known to be involved in the central processing of proprioception, a domain which we expect to be affected in frail individuals. The correct processing of proprioception is vital for everyday life activities and depends on the adequate processing of afferent sensory information and on its integration within coherent internal representations of the body (24). In this regard, the sensorimotor cortex, supramarginal gyrus (SMG), angular gyrus (AG), and superior parietal lobe (SPL) represent cortical structures consistently reported to engage in the processing of proprioception, spatial information, and internal representations (25–28). As a consequence, the study of FC alterations within these resting-state networks (RSNs) and regions of interest (ROIs) could carry particular relevance for the characterization of the neural correlates of frailty, more so since the electrophysiological signature of the syndrome remains greatly unexplored (16).

The aim of the present work was to investigate the existence of FC abnormalities within brain networks and structures involved in different aspects of motor function that might be affected in frail individuals. We used magnetoencephalography (MEG) to examine FC in the beta [12–30] Hz and alpha mu [8–13] Hz frequency bands, given the explicit association between those ranges of oscillation and sensorimotor function (29, 30). We hypothesized (1) that FC should be altered in frail individuals when compared to their robust counterparts in the above-mentioned RSNs and ROIs, and (2) that MEG is a useful tool to assess brain alterations in cohorts of cognitively healthy frail individuals. In definitive, this work is an attempt to increase our understanding of the electrophysiological implications of frailty and the role of the CNS in the development of the physical manifestations of the syndrome.

## Materials and Methods

### Participants

The sample of this study was recruited from an outpatient basis of the Geriatric Service of the Getafe University Hospital, Madrid. One hundred seventy candidates were originally contacted: for each frail candidate contacted, 2 robust participants of the same age (±1 year) and gender were included. We applied Fried's criteria (4) to assign each candidate into either the robust or the frail group. Participants were included in the frail group if meeting ≥3 out of the following criteria: unintentional weight loss (≥4.5 kg in the last year), decreases in gait speed, decreases in grip strength, self-reported exhaustion, and sedentary behavior. If none of these criteria were met, the participants were assigned to the robust group. To maximize the findings of our study bearing the limited sample size, pre-frail participants (participants meeting 1–2 of Fried's criteria) were not considered for inclusion. Finally, 60 right-handed older adults took part in the current study, all of them native Spanish speakers.

During the initial screening session, an expert neuropsychologist assessed the general cognitive status of the pre-selected sample with the Mini-Mental State Examination (MMSE). Participants scoring ≥24 in the MMSE underwent a more exhaustive neuropsychological evaluation examining additional cognitive domains: (1) Attention and executive functioning, evaluated with the Trail Making Test parts A and B (TMTA and TMTB) and the Direct and Inverse Digit Span test. (2) Memory, evaluated with the Rey-Osterrieth Complex Figure (ROCF) and the Logical Memory Score from the Wechsler Memory Scale-III. (3) Language function, evaluated with the Boston Naming Test (BNT) and the Phonemic and Semantic Fluency Tests from the Controlled Oral Word Association Test (COWAT). Additionally, we assessed the functional status of the participants using the Barthel Index for Activities of the Daily Living (ADL) and the Short Physical Performance Battery (SPPB). Neuropsychological, functional, as well as demographic information, is summarized in Table 1.

**Table 1:** Demographic, functional, and neuropsychological assessment data for each study group (robust and frail).

	**Robust**	**Frail**	***p*-value**
***n***	34	20	
**Age** median [IQR]	78.50 [74.00, 82.00]	81.00 [77.75, 82.75]	0.104
**Gender** *n* [% female]	21.00 [61.80]	15.00 [75.00]	0.381
**Functional status** median [IQR]
ADL	100.00 [100.00, 100.00]	92.50 [80.00, 100.00]	**<0.001**
SPPB	10.00 [9.00, 11.75]	7.00 [5.00, 8.00]	**<0001**
**Neuropsychological assessment** median [IQR]
**Memory**
Logic memory (I.A)	6.00 [5.00, 7.00]	5.00 [4.00, 6.25]	0.213
Logic memory (I.B)	7.00 [4.00, 10.75]	5.00 [4.00, 11.00]	0.739
Logic memory (II.A)	2.00 [0.00, 5.00]	0.50 [0.00, 3.25]	0.347
Logic memory (II.B)	5.00 [3.00, 7.75]	4.50 [0.75, 6.25]	0.427
ROCF (copy)	28.50 [25.00, 32.00]	29.00 [25.50, 31.00]	0.936
ROCF (immediate recall)	12.50 [8.62, 15.50]	8.50 [4.62, 12.50]	**0.039**
ROCF (delayed recall)	11.00 [8.50, 14.00]	8.00 [2.75, 11.62]	**0.021**
**Language**
BNT	15.00 [15.00, 15.00]	15.00 [14.00, 15.00]	0.298
Phonemic fluency	21.00 [14.00, 27.50]	20.00 [15.50, 25.00]	0.641
Semantic fluency	14.00 [11.00, 17.75]	10.50 [9.00, 14.25]	**0.045**
**Attention and executive function**
TMTA (time)	93.00 [69.25, 131.50]	116.50 [69.75, 195.50]	0.119
TMTA (errors)	0.00 [0.00, 1.00]	1.00 [0.00, 1.25]	0.175
TMTB (time)	307.50 [198.50, 379.00]	395.50 [282.00, 604.00]	0.130
TMTB (errors)	2.00 [1.00, 4.75]	4.00 [3.00, 6.00]	**0.032**
Direct digit	7.00 [6.00, 8.00]	6.00 [4.75, 7.00]	**0.036**
Inverse digit	6.00 [5.00, 7.00]	6.00 [5.00, 6.00]	0.424
**Cognitive status**
MMSE	27.00 [26.00, 28.00]	26.00 [25.00, 28.00]	0.543

*ADL, Barthel Index for Activities of the Daily Living; SPPB, Short Physical Performance Battery; ROCF, Rey-Osterrieth Complex Figure; BNT, Boston Naming Test; TMTA, Trail Making Test Part A; TMTB, Trail Making Test Part B; MMSE, Mini-Mental State Examination; IQR, Interquartile Range. Bold values indicate significant results*.

#### Inclusion and Exclusion Criteria

The following criteria were imperative for the inclusion of participants in the study: (1) Required age of at least 70 years. (2) Normal cognition as determined by a score ≥24 in the MMSE. (3) No history of psychiatric disorders (including Axis I disorders such as depression), neurological pathology, or drug consumption that could interfere with MEG. (4) Absence of medical conditions that could be associated with cognitive symptoms. (5) Absence of severe head injuries within the last 5 years. (6) Absence of usual alcohol intake (>3 alcoholic beverages per day). (7) Absence of sensory deficits (e.g., visual or auditive) that could prejudice the cognitive assessment.

Last, patients with a history of stroke were excluded only if showing focal neurological symptoms in the neurological exam or focal impairment on a magnetic resonance imaging (MRI) scan. Individuals with pacemakers or metallic implants that might interfere with MEG or MRI were also excluded. All the participants were free of ischemic cerebral lesions, a pathophysiological factor also reported to be involved in generating frailty without cognitive symptoms (31).

Out of the 170 candidates contacted, 56 refused to participate in the study and 14 candidates didn't contact back. One candidate had passed away. Ninety-nine participants were initially favorable to enrolling, however, 5 scored below the MMSE inclusion threshold. Ten presented pacemakers (1), metallic implants (6), prosthesis (1), or stents (2). One candidate was excluded due to severe vision impairment, one candidate was excluded because he was pre-frail and had been misclassified, two candidates could not walk, eight candidates were excluded due to their prescribed medication: deprax (1), diazepam (2), gabapentina (3), tapentadol (1), targin (1). Nine candidates were excluded or refused to participate due to diverse medical conditions: cancer (4), anemia (1), myocardial infarction (1), fibromyalgia (1), pharyngitis (1), and low back pain (1). Two candidates were excluded because they had family members in the study, and 1 candidate was excluded due to not matching the inclusion criteria after a stroke.

This study was approved by the Ethical Committee of the University Hospital of Getafe (Madrid). All the participants provided signed informed consent before enrolling in the study.

### MEG Recordings

Each participants' electromagnetic brain activity was acquired using a 306 channel (102 magnetometers, 204 planar gradiometers) Vectorview MEG system (Elekta AB, Stockholm, Sweden). This system is placed within a magnetically shielded room (VacuumSchmelze GmbH, Hanau, Germany) in the Laboratory of Cognitive and Computational Neuroscience of the Center for Biomedical Technology in Madrid. The MEG procedure was carefully explained to all the participants upon their arrival at the Center. We employed a three-dimensional Fastrak digitizer (Polhemus, Colchester, Vermont) to acquire the spatial outline of the headshape of the participants, the coordinates of three fiducial landmarks (nasion and bilateral preauricular points), and the coordinates of four Head Position Indicator (HPI) coils. The HPI coils were placed bilaterally over the forehead and the mastoids to monitor the position of the head during the recordings (for correction purposes in later processing stages). The preparation setup also included the placement of two electrooculogram electrodes (above and below the left eye) to record blinks and eye movements, and of two electrocardiographic electrodes (placed symmetrically across the chest) to capture cardiac activity. After the initial setup, all the subjects sat comfortably in the MEG system under dim light and were instructed to wakefully rest for 10 min: the first 5 min with their eyes open and staring at a white fixation-cross, and the remaining 5 min with their eyes closed. Brain activity was recorded using a sampling rate of 1,000 Hz with an online anti-aliasing band-pass filter between 0.1 and 330 Hz.

### MEG Signal Preprocessing

Eyes-closed resting-state MEG signals were first processed using a temporo-spatial filtering algorithm (tSSS) (32) (correlation window = 0.9, time window = 10 s). tSSS filtering reduces the contributions of external magnetic sources to the resulting MEG recordings and mitigates the effects of head movements in the scanner. Ocular, muscular, and jump artifacts were detected by a double-checked approach in which the automatic identification provided by the Fieldtrip toolbox (33) was afterward visually confirmed by a MEG expert. We used an algorithm based on independent components to remove the contributions of both ocular and electrocardiographic signals from our data. The remaining data were segmented in epochs of 4 s of artifact-free activity: 77.5 [12.1] trials (mean [SD]) in the frail group, 81.0 [13.1] trials in the robust group with no significant group-level effect regarding the number of trials (*p* = 0.33). Only magnetometers' data were used in the subsequent analysis (34).

### MRI Acquisition

Besides individual MEG recordings, each subject underwent a T1-weighted MRI acquisition in the Magnetosur Clinic in Getafe (Madrid) using a 0.23 T open configuration (C-shaped) Philips Panorama MRI scanner (TR/TE = 470/16 ms, flip angle 90°, 5 mm slice thickness, 256 × 256 matrix and FOV 270 mm). Individual T1-weighted MRI images were employed in the source reconstruction pipeline explained hereafter.

### Source Reconstruction

Artifact-free epochs were band-pass filtered in the following bands of interest: alpha mu [8–13] Hz, low beta [12–20] Hz, and upper beta [20–30] Hz. Preceding the filtering, the epochs were padded with 2 s (2,000 samples) of real signal on both ends to avoid edge effects within the data. The source model consisted of 2,459 sources placed in a homogeneous grid of 1 cm in the Montreal Neurological Institute (MNI) template, then linearly transformed to subject space by an affine transformation. The leadfield was calculated using a single-shell model generated from the individual T1-weighted MRI using Fieldtrip. Linearly Constrained Minimum Variance (LCMV) beamformers were employed to obtain the source-space time-series by using the computed leadfield and building the beamforming filters with the epoch-averaged covariance matrix.

### Functional Connectivity Calculation

Statistical coupling between all pairs of sources was estimated with the phase-locking value (PLV) algorithm. This algorithm is based on the assumption that the degree of non-uniformity of phase differences between two time-series should be a good estimator of their coupling (35).

(1) $$\begin{array}{l} PLV_{k,l}=\left|\frac{1}{T}\underset{t}{\sum }e^{-j\left(\varphi_{k}\left(t\right)-\varphi_{l}\left(t\right)\right)}\right| \end{array}$$

where φ_*k*_(*t*) and φ_*l*_(*t*) are the instantaneous phases of signal *k* and signal *l* at instant *t*, *T* is the number of temporal points per segment, and *j* is the imaginary unit. We calculated the PLV values (*PLV*_*k, l*_) using the time-series of each pair of sources (*k, l*) as the input. When computed for each participant and frequency range, this calculation resulted in a matrix of dimensions 2, 459 × 2, 459 × 54 × 3 (sources × sources × subjects × frequency bands). The elements of this matrix contain the PLV values for each pair of sources, per subject, and frequency band.

**Figure 1:**
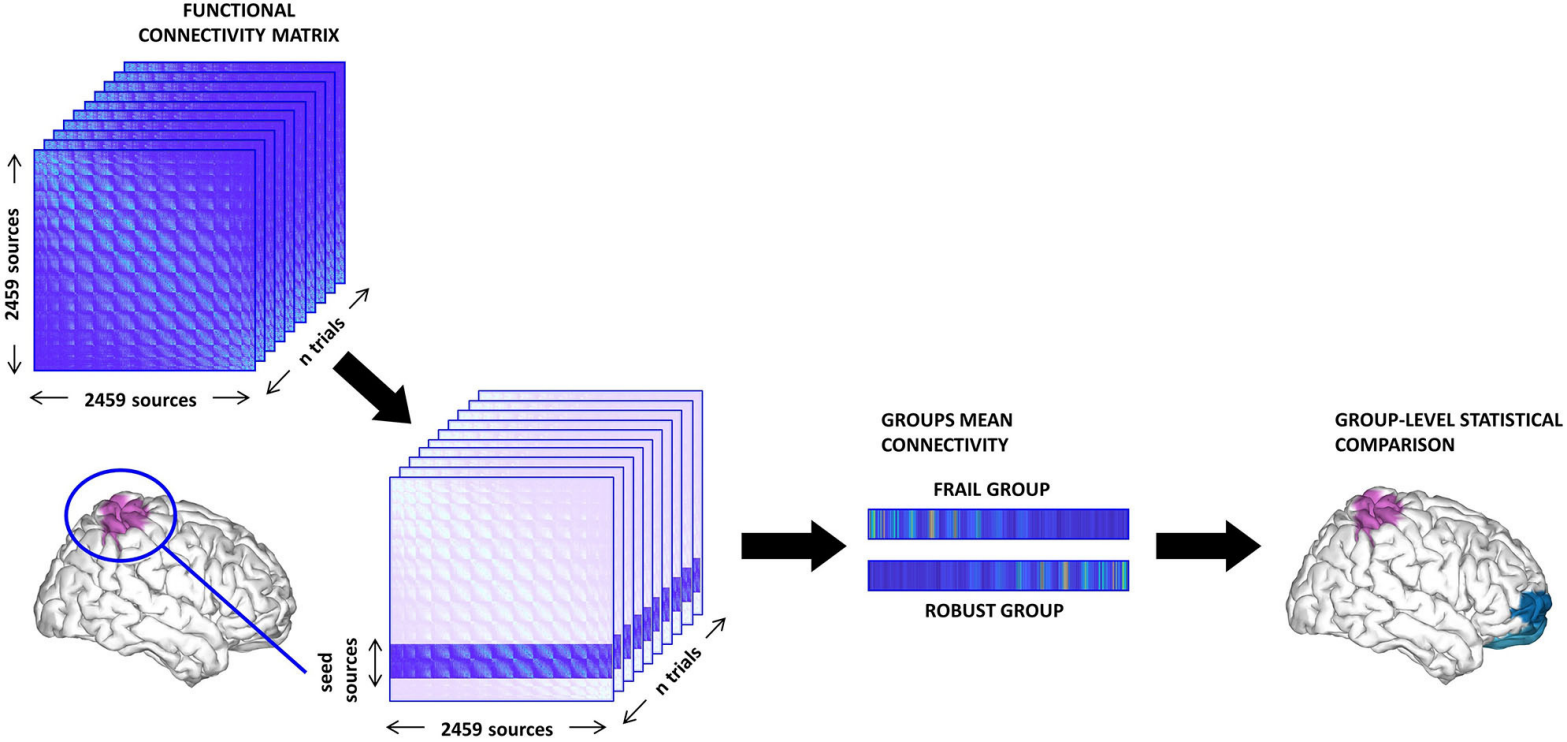
Schema of the seed-based functional connectivity (FC) analysis. First, the sources belonging to the seed are selected from the FC matrix. Then, we compute the mean value of the FC between the seed and the rest of the brain (excluding intra-seed pairs) and average across trials thus obtaining one vector per subject. Finally, we perform group comparisons and correct for multiple comparisons using a cluster-based method.

Two different analyses were performed using the PLV matrices:

1. *Seed-based analysis*. We tested whether the FC of relevant ROIs with the rest of the brain presented group differences. To this aim, we selected sets of cortical sources belonging to key areas for motor function and the central processing of proprioception [i.e., SMG, SPL, AG, and sensorimotor cortex (pre-central and post-central gyri)]. Then, the PLV values of all the sources within a particular seed to each individual distant source (intra-seed pairs were not considered) were averaged into a single vector representing the mean FC of the seed to each individual distant source. These values were compared between groups (see Figure 1). We studied left, right, and bilateral FC patterns separately for each of the aforementioned seeds. We used the Harvard-Oxford atlas for the anatomical parcellation of the ROIs.
2. *Resting-state network analysis*. We performed group comparisons of FC values in some RSNs relevant to the objectives of this study. Specifically, the synchronization levels of the posterior default mode network (pDMN), anterior default mode network (aDMN), DAN, ventral attentional network (VAN), FPN, and SMN were analyzed. We defined each RSN in accordance with previous literature, by selecting reported sets of ROIs represented by their MNI coordinates. The PLV values for all the sources included in a sphere of 1.5 cm radius around each MNI coordinate were averaged to obtain a single mean FC value for each RSN that was then compared between groups.

### Statistical Analyses

Age and gender were matched so that they did not differ between groups. In the seed-based analysis, independent samples *t*-tests were used to perform group comparisons of the mean FC between the seed and each distant source. The substantial number of links between the seed and the remaining cortical sources made it necessary to apply a method for multiple comparisons correction. To this aim, we performed a Montecarlo permutation procedure based on the grouping of significant sources that, if spatially contiguous, add up forming clusters that are compared in terms of their statistical size. The exact details of the cluster-based permutation tests (CBPT) method can be found in its original formulation (36). Furthermore, the mean FC of each RSN was compared between groups by using an independent samples *t*-test for each RSN and frequency band. For the statistical comparison of functional and neuropsychological scores, we used Fisher's exact test and the Mann-Whitney *U*-test for continuous variables.

## Results

Sixty participants were finally enrolled in our study, six of which were discarded for the FC analyses due to problems in the recorded MEG signals. In the final sample of 54 participants, 34 were robust, and 20 were frail. We did not observe significant group differences regarding age (*p* = 0.104) nor gender (*p* = 0.381). Frail participants demonstrated reduced functional levels as shown by the significantly lower scores attained on both ADL (*p* < 0.001) and SPPB (*p* < 0.001). In the executive domain, frail participants performed significantly worse in the TMTB (errors) (*p* = 0.032) and the Direct Digits Span test (*p* = 0.036). In the memory domain, frail participants attained poorer scores on both recall trials of the ROCF: immediate (*p* = 0.039) and delayed (*p* = 0.021). Finally, performance in the Semantic Fluency Test was also lower in the frail group (*p* = 0.045).

Among those filling Fried's criteria for frailty, only 1 participant fulfilled the five criteria, while 95% of frail participants met 3 (9; 45%) or 4 (10, 50%) criteria. Fatigue was the most prevalent criterion (95%), while weight loss (25%) was the least frequent (Table 2).

**Table 2:** Participants (number and percentage) in the frail group meeting each of the criteria of Fried's frailty phenotype.

**Fried's frailty phenotype criteria**	**Frail participants meeting each criterion *n* [%]**
Self-reported exhaustion	19 [95]
Involuntary weight loss	5 [25]
Reduced physical activity/sedentarism	8 [40]
Reduced gait speed	13 [65]
Reduced grip strength	17 [85]

### Seed-Based Analysis

FC patterns of relevant regions were compared between groups. Three significant clusters of FC differences arose after multiple comparisons correction: two belonging to seeds located in the right hemisphere (AG and SPL) and one involving the bilateral SMG. FC disruptions demonstrated a consistent direction: in all cases, the frail group exhibited reduced synchronization between the seeds and the clusters in the upper beta frequency band.

**Figure 2:**
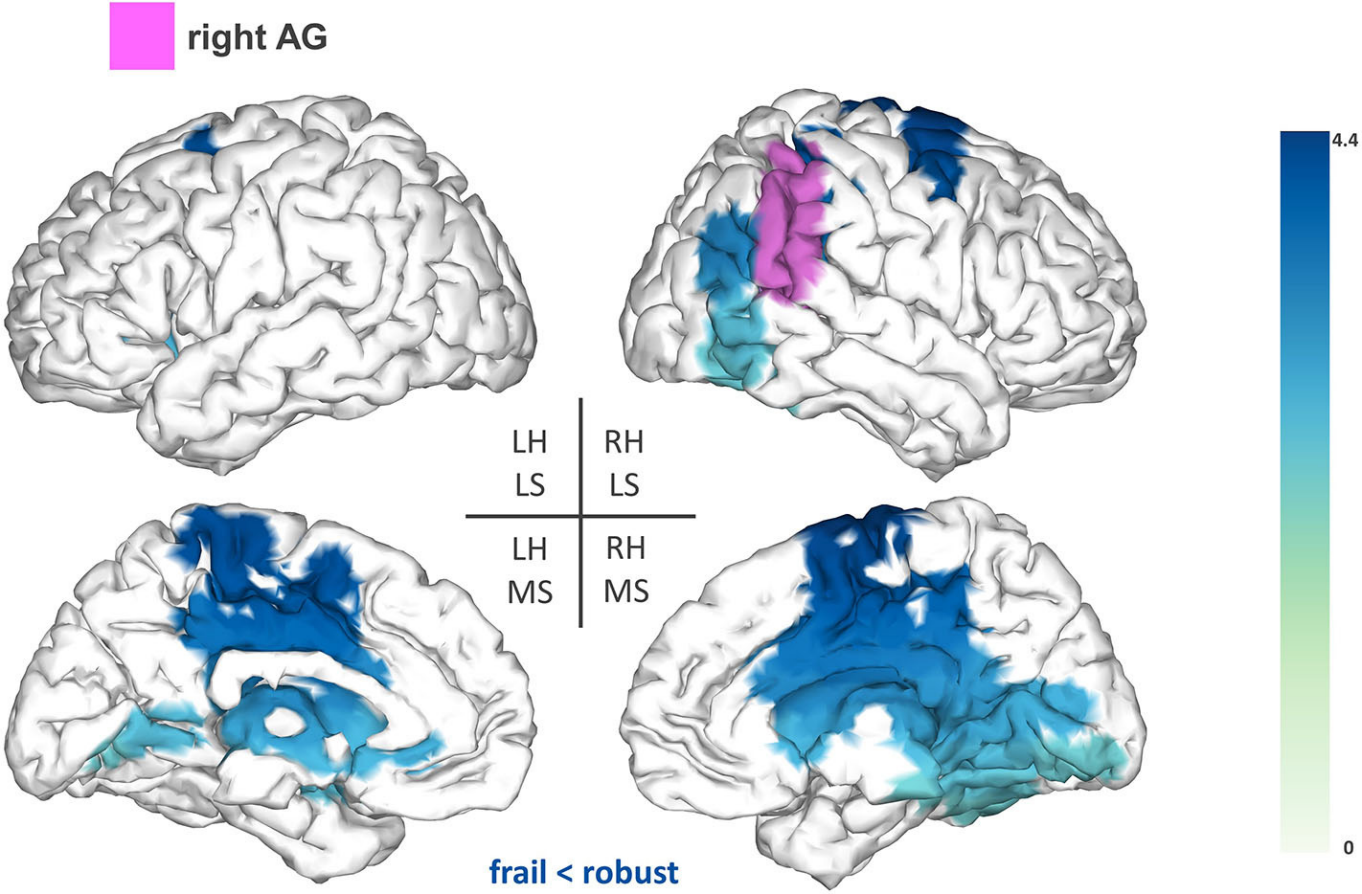
Seed-based functional connectivity analysis results for the right angular gyrus (rAG). The pink area represents the seed. The areas shaded in blue outline the clusters that are significantly hypoconnected to the rAG in frail individuals when compared to robust participants. The blue colormap indicates hyposynchronization in the frail group between the seed and the cluster. The blue colorbar represents the *t*-statistic values of each source of the significant cluster obtained by independent samples *t*-test. RH, right hemisphere; LH, left hemisphere; LS, lateral surface; MS, medial surface.

Firstly, we found reduced FC between the right AG (T-statsum = 477.06, *p* < 0.05) and widespread regions including the right motor cortex, precuneus, posterior cingulate cortex, and the sensorimotor cortex (pre-central and post-central gyri) (Figure 2).

**Figure 3:**
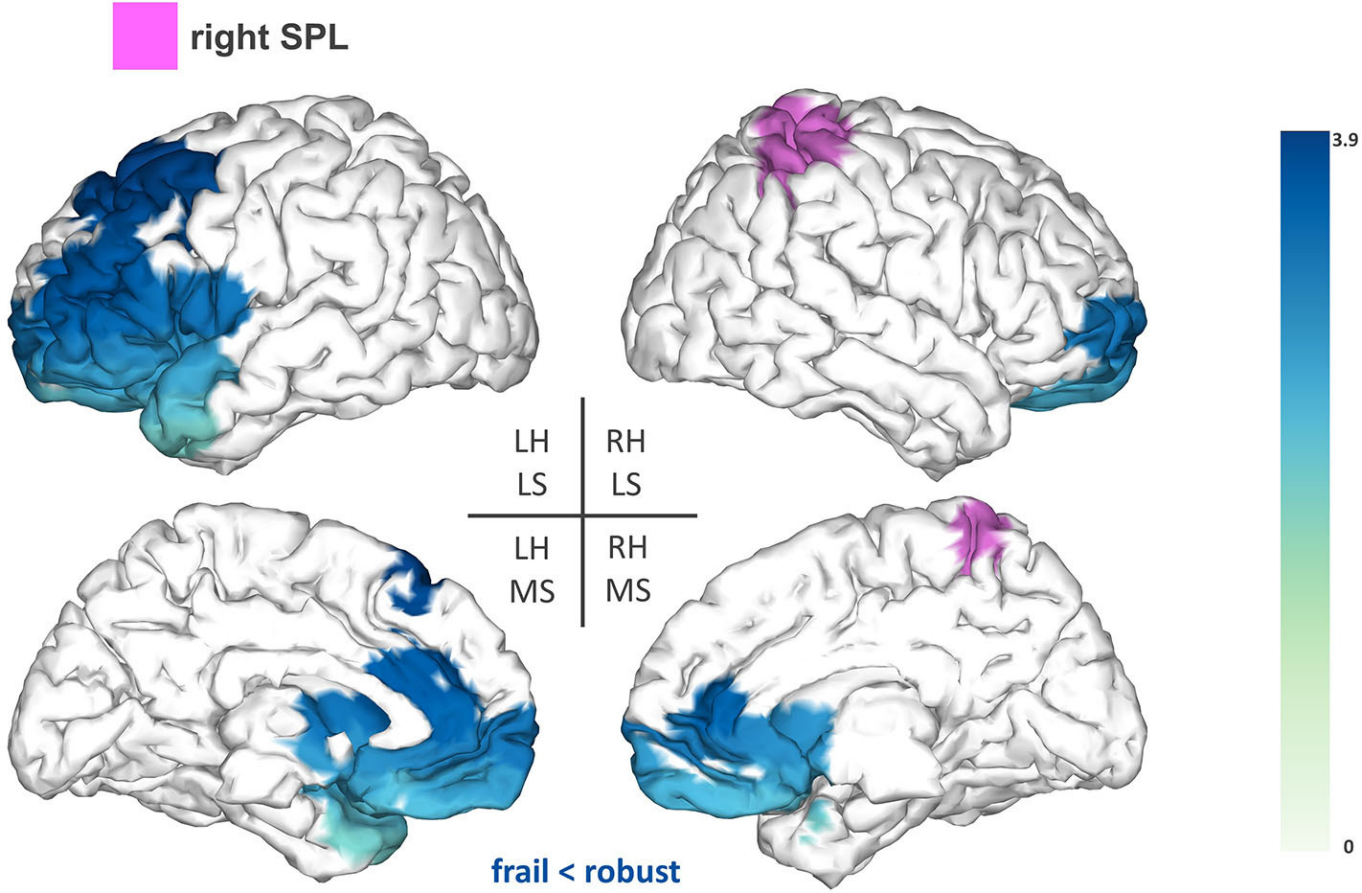
Seed-based functional connectivity analysis results for the right superior parietal lobe (rSPL). The pink area represents the seed. The areas shaded in blue outline the clusters that are significantly hypoconnected to the rSPL in frail individuals when compared to robust participants. The blue colormap indicates hyposynchronization in the frail group between the seed and the cluster. The blue colorbar represents the *t*-statistic values of each source of the significant cluster obtained by independent samples *t*-test. RH, right hemisphere; LH, left hemisphere; LS, lateral surface; MS, medial surface.

Secondly, we found reduced FC between the right SPL (T-statsum = 608.7, *p* < 0.05) and anterior regions of the left hemisphere such as lateral and medial frontal areas (e.g., premotor cortex), the frontal pole, the anterior cingulate, and the left temporal pole (Figure 3).

**Figure 4:**
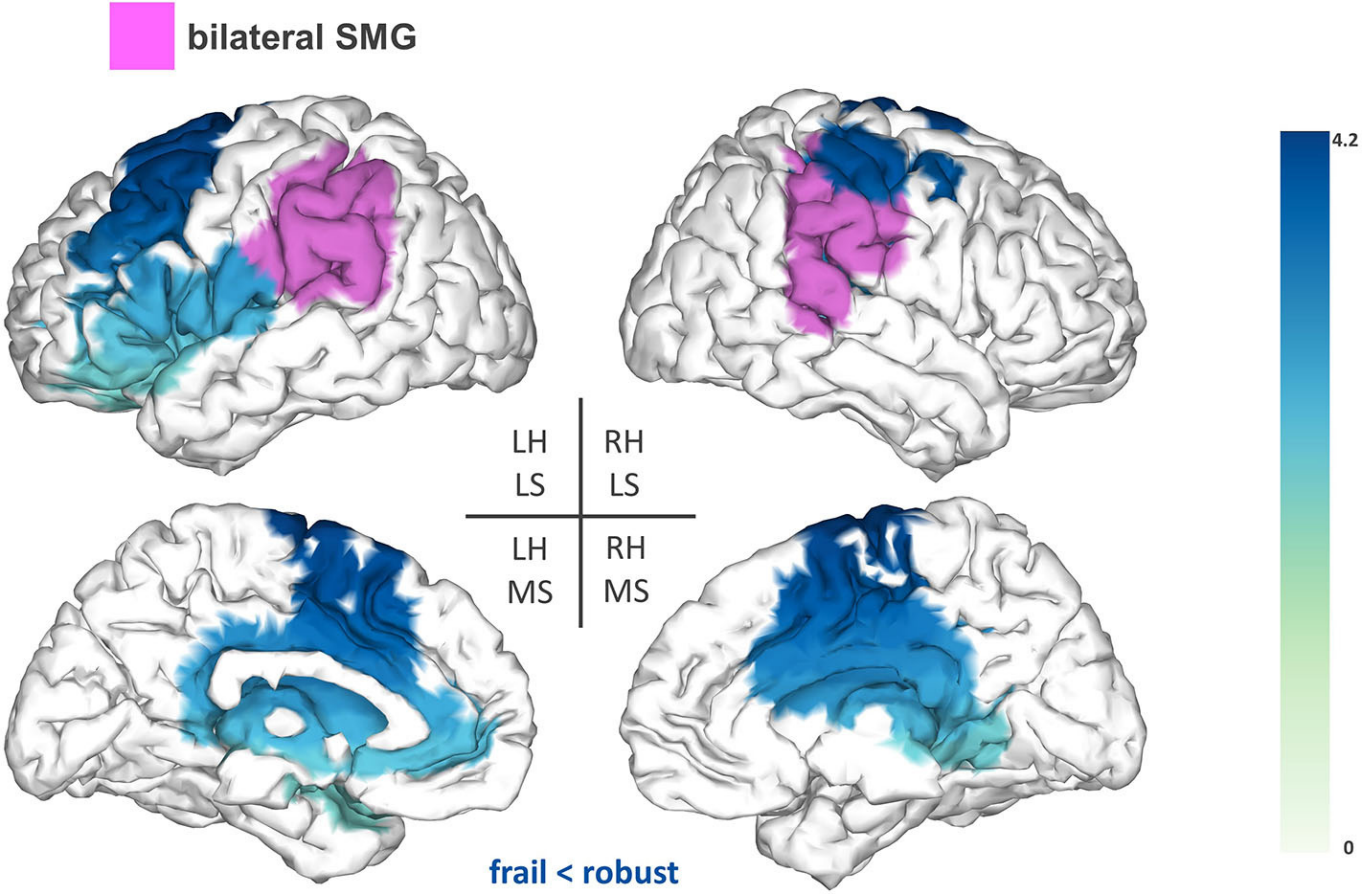
Seed-based FC analysis results for the bilateral supramarginal gyrus (SMG). The pink area represents the seed. The areas shaded in blue outline the clusters that are significantly hypoconnected to the bilateral SMG in frail individuals when compared to robust participants. The blue colormap indicates hyposynchronization in the frail group between the seed and the cluster. The blue colorbar represents the *t*-statistic values of each source of the significant cluster obtained by independent samples *t*-test. RH, right hemisphere; LH, left hemisphere; LS, lateral surface; MS, medial surface.

Lastly, the bilateral SMG showed reduced FC (T-statsum = 713.2, *p* < 0.05) to the left premotor cortex [inferior frontal gyrus (IFG) and middle frontal gyrus (MFG)], the supplementary motor area (SMA), areas of the sensorimotor cortex, the right precuneus, and the bilateral cingulate cortex (Figure 4).

We could not find significant differences when the sensorimotor cortex was used as a seed, nor for the alpha mu and low beta frequency bands.

### Resting-State Network Analysis

Frail older adults showed an overall reduced intra-network mean FC when compared to their robust counterparts. More concretely, frail participants exhibited reduced FC in the upper beta band within the FPN (*p* = 0.028), pDMN (*p* = 0.042), and VAN (*p* = 0.039). Furthermore, the DAN showed a strong trend (*p* = 0.066) in the same direction (i.e., toward lower FC in the frail group) also in the upper beta band.

## Discussion

Previous findings suggest that disturbed brain FC could contribute to the decline in motor function observed in frail individuals. In the present work, we used MEG to compare the FC patterns of robust and frail participants without global cognitive impairment. In the frail group, FC was significantly reduced between posterior regions of the parietal cortex (i.e., SMG, rSPL, and rAG) and fronto-parietal clusters of brain areas relevant for motor function. Furthermore, intra-network mean FC was also significantly reduced in the frail group within RSNs of interest such as the FPN, pDMN, and VAN.

Group comparisons of the seed-based analysis revealed significant FC reductions in the frail group for the following seeds: SMG, rSPL, and rAG. The seed reference regions of our study were chosen as we expected that alterations in the central processing of proprioception could contribute to some of the motor deficits manifested by frail individuals. Proprioception is defined as the ability to integrate sensory signals from multiple mechanoreceptors (i.e., muscle spindles, joint receptors, skin stretch receptors) to sense the position and movement of our body in space (24). The correct processing of proprioception is pivotal for motor control and relies on the detection and transmission of afferent information, as well as on its adequate integration within the context of updated internal representations of the body (i.e., the body schema) (24). High-order parietal cortices have been linked with the central processing of proprioception (37), notably the SMG (25). In our study, frail individuals demonstrated reduced FC between the SMG and a fronto-parietal cluster including the left premotor cortex (IFG and MFG), SMA, cingulate cortex, and areas of the sensorimotor cortex. Different fMRI studies have reported proprioceptive-related activations of the SMG alongside several of the sensorimotor and fronto-parietal areas that were functionally hypoconnected to the SMG in our study (25, 26, 38). This evidence substantiates the existence of a functional association between those areas for the central processing of proprioception, which might be undermined in frail individuals as hypothesized from the reduced FC associations observed herein.

Additionally, frail participants exhibited reduced FC between the rSPL and frontal areas of the left hemisphere (e.g., premotor cortex and frontal pole). Several studies have suggested that the SPL plays a role in sensorimotor integration, specifically, in the maintenance of updated representations of the body schema (27, 39, 40). These studies are based on the hypothesis that the brain builds internal “forward models” to estimate the consequences of a motor command, and in turn, to anticipate the postural configuration that will arise after an upcoming movement. This recursive process provides continuously updated representations of the body schema, that are reportedly maintained within the SPL to be used during the planning and execution of a movement (27, 39, 40). Reduced FC between the rSPL and frontal areas involved in different aspects of motor planning (41, 42) might compromise the preparation of a movement in compliance with accurate estimates of limb position, increasing the risk of imprecise or ill-fated movements in the frail population. This possibility is consistent with previous results from Parkinson et al. showing that activity within the premotor cortex influences BOLD activation in the rSPL in relation to the coding of upper limb posture (40). Similar implications might arise from the reduced FC between the rAG and fronto-parietal regions (e.g., motor cortex and cingulate cortex) considering the engagement of the rAG in further aspects of body representation (28). Overall, our results indicate that frail individuals seem to demonstrate reduced FC within central structures mediating proprioceptive-related processes, which might account for deficiencies in motor control within this population. Nevertheless, additional task-based research is necessary to further elucidate this association.

The connection between reduced FC and impaired performance discussed herein is in agreement with the still scarce literature exploring brain dysfunction in frail cohorts (16). Only one previous study directly addressed the correspondence between FC and frailty, using resting-state fMRI and Fried's modified frailty score (21). Lammers et al. found reduced FC in frail individuals within a fronto-parietal network including the SMA. In their study, spontaneous BOLD fluctuations in the SMA correlated with BOLD activation in the anterior cingulate and the sensorimotor cortex, supporting the idea of a common functional relationship between some of the areas that showed reduced FC in our study. Importantly, FC within this network correlated with neurocognitive measures of motor speed and manual dexterity. Complementarily, several studies documented associations between individual items used to assess frailty (e.g., gait speed, grip strength) and FC measures in cohorts of healthy older adults. Seidler et al. (15) explored the relationship between grip strength and sensorimotor FC, reporting a generalized association of increased FC with enhanced grip performance. Similarly, Yuan et al. (14) demonstrated that gait speed under demanding conditions was associated with greater frontal FC in comparison to gait speed during a simpler task. Overall, previous research seems to concur in the general idea that reduced FC negatively impacts different aspects of motor performance in older adults. On a separate note, it is important to mention that FC reductions in our results consistently concerned upper beta frequencies, which have been classically linked to motor function (30). At the same time, beta-band oscillations have been proposed to play a role in sensorimotor integration that is of particular relevance in the context of our study. According to this hypothesis, beta-band oscillations may ensure the efficient processing of proprioceptive information that is required for recalibrating the sensorimotor system in preparation for upcoming movements [reviewed in (43)].

The analysis of RSNs revealed additional FC reductions in the frail group for upper beta frequencies. Frail individuals showed reduced intra-network FC within the FPN, pDMN, and VAN. Reduced FPN FC was rather anticipated considering the involvement of the FPN in motor planning (44) and executive processes relevant for motor control (45, 46). Lo et al. (23) reported an association between higher FPN FC and faster gait speed, which they interpreted as a dependence of gait integrity upon the proper communication of executive brain areas within the FPN. This result fits in with the lower FPN FC found in our frail sample, particularly in light of the close relationship between frailty and slowing gait speed (47) that has led to include a 20% reduction in gait speed among Fried's frailty phenotype (4). This study also revealed an attentional effect over gait steadiness, characterized by the correlation of gait variability with weaker negative FC between the DMN and the DAN (23). Since we did not explore inter-network FC, we could be missing additional disruptions in attentional modulation that might be influenced by the reduced FC seen within the VAN and the DAN. Finally, we observed reduced FC within the pDMN in frail individuals. The pDMN comprises two prominent nodes of the DMN, namely the precuneus and the posterior cingulate cortex (48). Both areas are believed to engage in integrating information from multiple sources into additional aspects of spatial and self-representation (49). Accordingly, reduced synchronization within the posterior nodes of the DMN might further impact the processing of internal representations in frail individuals.

To our knowledge, this study constitutes the first report in the literature of frequency-specific alterations in the FC patterns of frail older adults. A potential shortcoming of our study is the inclusion of a limited sample that was sourced from a single study setting, even though this also guarantees homogeneity in the clinical assessment of frailty. It is important to note that the multifactorial etiologies of frailty cannot be resumed by the study of a single system, the CNS in our case, but demand the integration of physiological models of diverse interacting systems. For example, some of the proprioceptive-related outcomes predicted by frailty (e.g., recurrent falls, decline in daily activities, reduced gait speed) could partly originate in the muscular periphery as a consequence of age-related disruptions in muscular integrity (i.e., sarcopenia). Aging is accompanied by changes in muscle spindle composition, that possibly affect motor innervation, and in turn, the effective transmission of sensory signals for motor control (50, 51). At the same time, aging also comes with changes within central structures that mediate in the processing of proprioceptive information (52). In consequence, proprioceptive-related failures might represent the joint expression of age-related changes within central and peripheral structures. Following this, our results suggest that some of the motor deficits associated with frailty are possibly not only due to peripheral factors like sarcopenia but to additional perturbations within relevant central structures. Taken as a whole, our study demonstrates the presence of abnormalities in the upper beta FC patterns of frail individuals free of cognitive impairment and ischemic cerebral lesions.

## Data Availability Statement

The datasets generated for this study are available on request to the corresponding author.

## Ethics Statement

The studies involving human participants were reviewed and approved by the Ethical Committee of the University Hospital of Getafe (Madrid). The patients/participants provided their written informed consent to participate in this study. Written informed consent was obtained from the individual(s) for the publication of any potentially identifiable images or data included in this article.

## Author Contributions

IS-M carried out the MEG studies, the analysis of the data, and the writing of the first draft and the final version. SD contributed to the analysis of the data and the writing of the first draft and the final version. SW designed the study, coordinated its implementation, and made substantial contributions to the analyses and writing of the manuscript. NP, RB, and MV were responsible for the recruitment of the participants, their functional and clinical phenotyping, the design of the psychological assessment, and the clinical groundwork and logistics. EG was involved in interpreting the MRI images. FM, DL-S, and LR-M were involved in the conception, design, supervision, and analysis of the data. They also participated in the writing of the draft versions and in the writing of the final version of the manuscript. All authors read and approved the final manuscript.

## Conflict of Interest

The authors declare that the research was conducted in the absence of any commercial or financial relationships that could be construed as a potential conflict of interest.
